# Effects of occlusal splint and exercise therapy, respectively, for the painful temporomandibular disorder in patients seeking for orthodontic treatment: a retrospective study

**DOI:** 10.1186/s12903-022-02538-y

**Published:** 2022-11-24

**Authors:** Junjie Chen, Ruoyu Ning, Yanqin Lu

**Affiliations:** 1 Department of Orthodontics, Xiangya Stomatological Hospital & Xiangya School of Stomatology, Hunan Clinical Research Center of Oral Major Diseases and Oral Health, Central South University, Changsha, Hunan Province China; 2 Department of Orthodontics, Third Xiangya Hospital & Xiangya School of Medicine, Central South University, Changsha, 410008 Hunan China

**Keywords:** HSS, Orthodontic treatment, Painful TMD, Disc-condyle position

## Abstract

**Objective:**

To evaluate the effect of hard stabilization splints (HSS), counselling and exercise therapies, respectively, for the painful temporomandibular disorder (TMD) in patients seeking for orthodontic treatment through magnetic resonance imaging (MRI) and clinical examination.

**Materials and methods:**

Eighty-seven TMD patients were divided into two groups according to their therapies: the HSS group (*n* = 43) comprising of patients treated with HSS, counselling and masticatory muscle exercises; the control group (*n* = 44) comprising of patients treated with counselling and masticatory muscle exercises alone. All patients had orthodontic therapies after the first treatment phase. The joint pain and clicking of all patients were recorded via clinical examination. MRIs of HSS groups were taken before (*T*_0_), after the first phase (*T*_1_), and after the orthodontic treatment (*T*_2_). Parameters indicating the condyles and articular discs were evaluated. Clinical symptom (pain and clicking) changes among *T*_*0*_, *T*_*1*_ and *T*_*2*_ time point were detected in the two groups respectively. The significant differences between HSS and control groups, as well as between male and female were tested at *T*_*1*_ and *T*_*2*_. Position changes of condyles and discs in HSS group among *T*_*0*_, *T*_*1*_ and *T*_*2*_ were detected in male and female respectively.

**Results:**

After the first treatment phase, there was no difference in the decrease of facial pain between the two group, as well as between male and female in the two groups (*P* > 0.05). Clicking decreasing was not statistically significant. After the whole orthodontic periods, the TMJ pain relapsed in female of the control group, and the number of female’s pain joints was more than male’s (*P* < 0.05). In the HSS group, the posterosuperior movements of discs and the anteroposterior movements of condyles were recorded in closing position (*P* < 0.05). After the whole orthodontic periods, female’s disc-condyle angles increased, the discs to HRP distance decreased and condyles to VRP distance increased when compared with the data of *T*_1_ (*P* < 0.05).

**Conclusions:**

For the orthodontic patients with painful TMD, HSS combined with counselling and exercise therapies before orthodontic treatment could provide pain relief. HSS is helpful to improve the position and relation of discs and condyles. In addition, male's prognosis is better than female's in terms of stability.

## Introduction

Temporomandibular disorder (TMD) is a common condition and frequently encountered disease in oral system, which is a general term for a group of diseases involving masticatory muscles, peripheral nervous system, and temporomandibular joints (TMJs) [1]. Main symptoms of these diseases are pain, joint friction, irregular or limited mandibular function, and they are more common in young female [2]. Anterior disk displacement (ADD) is one kind of TMD, including anterior disc displacement with reduction (ADDR) and anterior disk displacement without reduction (ADDWR). Some pointed out that ADDR is the most frequent type of disc displacement [3, 4], and most TMD patients’ condyles were located in a posteroinferior position [5, 6].

The pathogenesis of TMD has not been fully clarified, which is related to psychosocial factors, immunity, occupational strain, TMJ overload and its anatomical factors and so on [7]. Occlusal factor plays a controversial role in the occurrence and development of TMD, which has been studied by numbers of researchers [8, 9]. But those thought occlusal factor as the most important are pretty old ones from mainly 20–40 years ago [10]. Current literatures have suggested to administrate TMD as a multifactorial problem, as some systemic or local factors such as cervical spine disorders, oral parafunctions [11], nicotine, sleep bruxism [12], and mental state [13] promote the development and progression of TMD.

The conservative therapy of TMD mainly include occlusal splint therapy, counselling, exercises, massage, manual therapy, which are considered as the first choices treatment for TMD pain because of their low risk of side effects [14]. Exercises therapy is one of effective treatment methods in rehabilitation to facilitate normal movement patterns through the biofeedback mechanism. Florjanski [15] included 10 papers into a systematic review, showed a significant correlation between biofeedback usage and reduction of masticatory muscle, that means exercises and biofeedback can be effective tools in painful TMD management. Hard stabilization splints (HSS) are also used to relax masticatory muscles and guide the mandible to a stable position, and they can effectively reduce clinical symptoms with the advantages of easy adaptation and simple preparation [16, 17].

The reality is that many patients seeking for orthodontic treatment do have some TMJ clinical symptoms, such as pain, friction and clicking. In orthodontic clinics, in order to alleviate the clinical symptoms of some patients with obvious TMJ pain, sometimes HSS, counselling or exercises therapy are used before orthodontic treatment to relieve TMJ symptoms and reduce TMJ discomfort [18, 19]. However, HSS usually causes more discomfort than exercise and counselling therapies. For the same purpose of alleviating clinical symptoms, would HSS therapy before orthodontic treatment be more effective than counselling and exercise therapies in alleviating pain, clicking? How about its effect on discs and condyles? Is there any difference about the efficacy between male and female?

The whole dynamic process from HSS, counseling, exercise therapies to the end of the occlusal reconstruction about TMJ symptoms are relatively rare. The aim of this study was to explore whether HSS combined with counselling and exercises has additional benefit in relieving TMJ pain, whether HSS will increase long-term efficacy stability in the following orthodontic treatment, to explore what the HSS dose to disc-condyle position. The null hypothesis is that no difference in efficacy would be found between HSS therapy and counselling, exercise therapies, as well as between male and female. This study could provide some references clinically.

## Materials and methods

### Participants

This retrospective study selected 20 to 30-year-old adults with painful TMD as the experimental object, and was done at the Xiangya Stomatological Hospital of Central South University with 41 males and 46 females. The samples were consecutive patients with detailed and complete clinical data who started the treatment after 04–2020 and ended before 06–2020. Subjects were selected according to the following criteria: (1) Meeting the diagnostic criteria for TMD research (RDC/TMD) [20], (2) unilateral or bilateral chronic TMJ pain in articulatory areas or masticatory muscles, no acute pain, (3) no degenerative disease on TMJ or oral parafunctional activity, (4) no trauma history or TMJ surgery history, (5) no orthodontic treatment history or restorations in the oral cavity, (6) no other systemic disease or clinical history.

### Treatment design

**Fig. 1:**
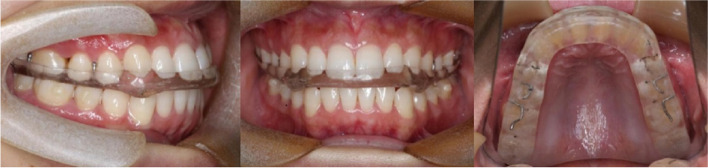
Hard stabilization splint on upper dental arch

Centric relation (CR) occlusion was performed by one hand induction directly by the same clinician and recorded by Delar wax. Then, occlusion records were transferred to the Germany SAM articular and the splints were produced on this CR position (Fig. 1).

Patients of HSS group were asked to wear HSS throughout night plus 4 h during the day, being 2 h in the morning and 2 h in the afternoon for 4 months. Clinicians have the HSS adjustably grinded on each subsequent visit (returning to the hospital twice a month in the first month, once a month afterward). In addition, counselling and masticatory muscle exercises were also implemented during the period. The control group implemented counselling and masticatory muscle exercises alone.

Fixed orthodontic treatment was used to all patients after the first treatment phase. Class I occlusion was achieved at the end of the orthodontic therapy and modified Hawley’s mechanical retainers were used for each patient. The implementation of treatment for all patients was performed by one clinician.

### Radiographic data

In the section of radiographic data, Authors should add the information weather or not the patients wore, as this, see:

- MRIs of three different stages in the whole treatment procedures including pre-splint (*T*_*0*_), immediate post-splint (*T*_*1*_) and post-orthodontics (*T*_*2*_) of the patients in HSS group were accessed with closings and the greatest openings respectively. All MRI data were calibrated and obtained by the same observer with patients not wearing fixed retainers, as retainers may blur and cause distortions to the image [21]. MRIs were acquired by a 1.5-T GE Signa Ercite II scanner, America, with exposure settings 110 kV for T1W1 and T2W1. Oblique sagittal slices were obtained at FOV 12 mm; slice thickness, 2 mm; repetition time (TR), 200 ms; echo time (TE), 24 ms; scan time, 58 s; 256 × 256 pixel matrix; oblique coronal slices were obtained at FOV 20 mm; slice thickness, 2 mm; TR, 475 ms; TE, 24 ms; scan time, 58 s; 192 × 2560 pixel matrix. Three experienced radiologists evaluated all of the MRI images in a consensus approach.

**Fig. 2:**
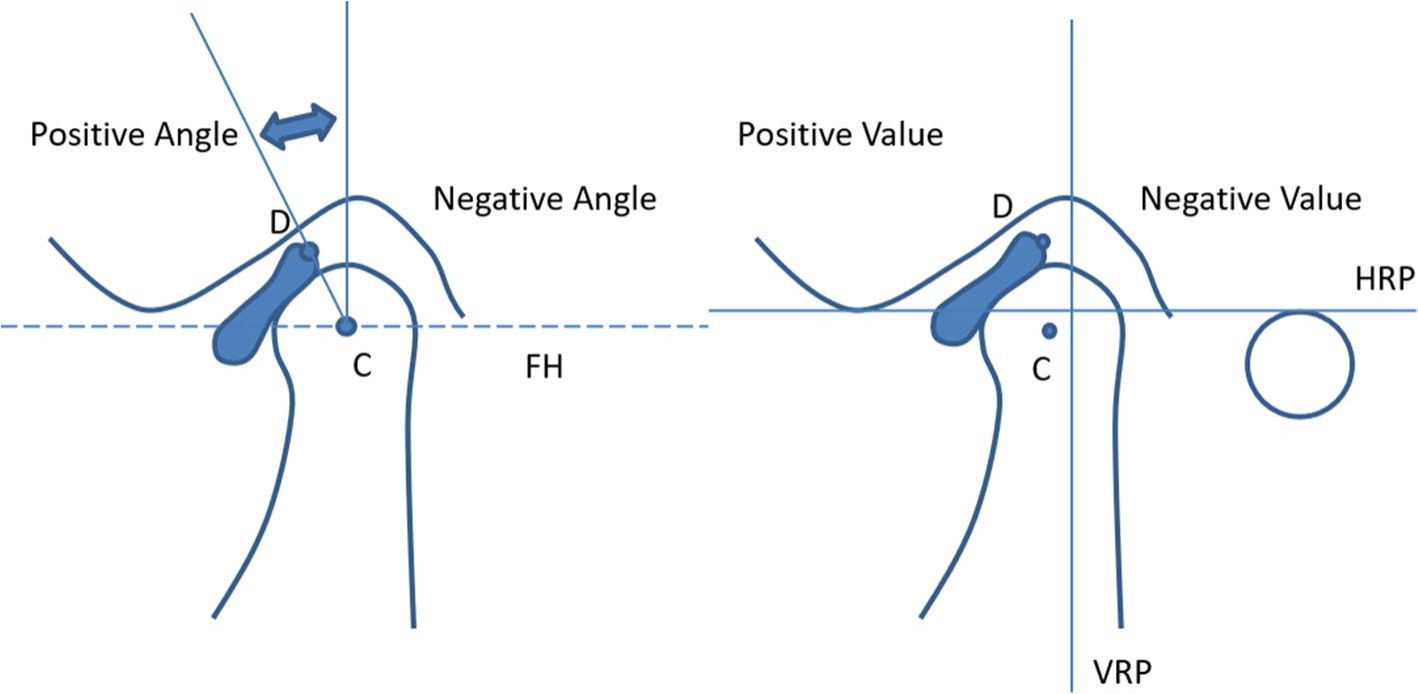
Illustration of the condyle-disc angle and position of condyles and discs. C point: the center of the condyles, D point: the posterior marginal midpoint of posterior articular disc band, FHVL line: the line perpendicular to the FH line at C point, ∠CD-FHVL: disc-condyle angle.

The midsagittal positions of condyles on MRIs were selected for image depiction and fixed point, and Drace-Enzmann’s method [22] was adopted for measurement. Descriptions on MRIs were designed to measure the position of condyles and discs, as well as the disc-condyle relations (Fig. 2). Detailed definitions and methods of each measurement are shown in Table 1 and Fig. 2.

**Table 1:** Definition of measurements

Measurements	Definition
MRI	
C point	the center of the condyles
D point	the posterior marginal midpoint of posterior articular disc band
FHVL line	the line perpendicular to the FH line at C point
∠CD-FHVL(°)	disc-condyle angle, whose normal range was from -10°to 10°, and any more than 10° was defined as ADD
C-HRP(mm)	the distance between C point and the horizontal reference plane (drawn by connecting the lowest point of articular nodule and the uppermost point of external auditory canal)
D-HRP(mm)	the distance between D point and the horizontal reference plane
C-VRP(mm)	the distance between C point and the vertical reference plane (drawn perpendicular to HRP at the highest point of articular fossa)
D-VRP(mm)	the distance between D point and the vertical reference plane

### Clinical efficacy evaluation

**Fig. 3:**
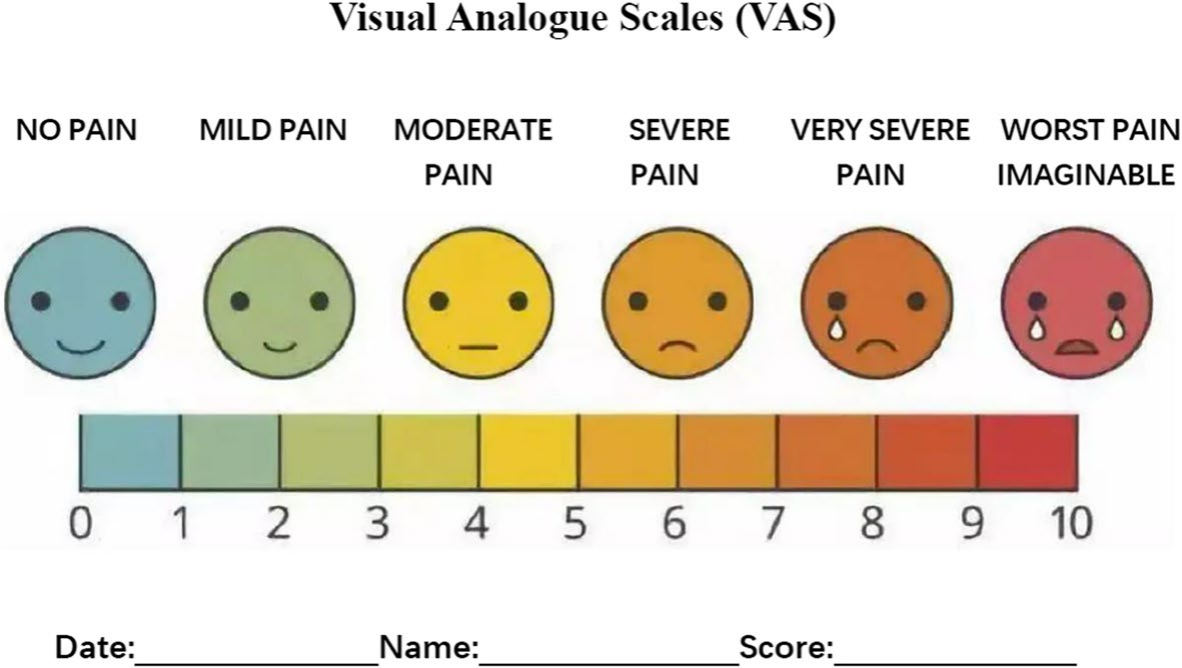
Visual analogue scales (VAS) for patients

TMJ clicking: Whether there was joint clicking and the number of joints with clicking at *T*_*0*_*, T*_*1*_ and *T*_*2*_ were recorded. No clicking was defined as the disappearance of joint clicking during the whole opening and closing movement. (2) TMJ pain: The degree of TMJ pain, including masticatory and joint area, was assessed for signs and symptoms according to Mehra and Wolford (7) and Kurita et al. [23] Visual analogue scales (VAS) were used for subjective evaluation of joint pain (0 = no pain, 10 = severe pain) (Fig. 3). The TMD symptom assessments after treatment were done by one evaluator who was blinded to the history of each subject.

### Statistical analysis

All data were analyzed by IBM SPSS Statistics 21 software. Kolmogorov–Smirnov analysis and Fanchazzi analysis were used for the homogeneity of variance test and normal distribution, and statistical significance was established at α = 0.05. One- and two-way analysis of variance (ANOVA) with Tukey tests was used to detect clinical symptom changes among *T*_*0*_, *T*_*1*_ and *T*_*2*_time point. An independent sample t-test was used to test significant differences between HSS and control groups, as well as between male and female. To assess the reliability of these measurements, 30 subjects were randomly chosen. All measures were duplicated by the investigator R.N. An intraclass correlation coefficient (ICC) was used to determine the intraobserver reliability of the measurements through reliability analysis in SPSS. Reliability was divided into three categories: poor (ICC < 0.40), fair to good (0.40 ≤ ICC ≤ 0.75), and excellent (ICC > 0.75) [24].

## Reusults

Kolmogorov–Smirnov analysis and Fanchazzi analysis with all *P* < 0.05 shows that each set of data conforms to homogeneity of variance test and normal distribution. And the intra-observer reliability of the measurements of all descriptions using SPSS software was excellent, with ICC’s ranging from 0.821 to 0.898. The retrospective power was from 0.791 to 0.932.

### Distribution of diagnostic subgroups of TMD (Table 2)

**Table 2:** Proportion (%) of TMD diagnoses before treatment, assessed with the RDC/TMD criteria

Condition	HSS group (*n* = 43) No. of joints (% within group)	Control group (*n* = 44) No. of joints (% within group)
Myofascial pain	68(80)	70(80)
Myofascial pain with limited opening	1(1)	2(2)
Disc displacement with reduction		
Left	20(47)	21(38)
Right	18(42)	23(52)
Disc displacement without reduction		
Left	3(7)	2(5)
Right	4(9)	5(11)
Disc displacement without reduction, with limited opening		
Left	0(0)	0(0)
Right	0(0)	0(0)
Arthralgia		
Right extra-auricular	10(23)	9(20)
Left extra-auricular	8(19)	7(16)
Right intra- auricular	2(5)	1(2)
Left intra- auricular	1(2)	1(2)

The subjects in both the HSS and control groups that were examined according to RDC/TMD criteria showed none of the patients were diagnosed with Disc displacement without reduction, with limited opening.

### Comparison of clinical symptoms between HSS group and control group (Tables 3, and 4)

**Table 3:** ANOVA and Tukey LSD analysis for clinical symptoms of 87 patients (142 joints) (*P* < 0.05)

measurements	HSS group (*n* = 43)				Control group (*n* = 44)			
	*T*_*0*_	*T*_*1*_	*T*_*2*_	*P*	*T*_*0*_	*T*_*1*_	*T*_*2*_	*P*
TMJ Pain (VAS)	5.45 ± 1.42	1.47 ± 0.89	1.52 ± 0.75	0.001	5.27 ± 1.39	1.58 ± 0.74	3.84 ± 1.29	0.001
TMJ Pain (No. of joints)	60/70	10/70	15/70	0.001	62/72	13/72	45/72	0.001
TMJ Clicking (No. of joints)	37/70	32/70	36/70	0.557	40/72	37/72	35/72	0.278
Measurements	HSS group (*n* = 43)				Control group (*n* = 44)			
	T_0_ VS T_1_	T_1_ VS T_2_	T_0_ VS T_2_		T_0_ VS T_1_	T_1_ VS T_2_	T_0_ VS T_2_	
	*P*				*P*			
TMJ Pain (VAS)	0.001*	0.229	0.004*		0.002*	0.002*	0.493	
TMJ Pain (No. of joints)	0.001*	0.368	0.003*		0.005*	0.001*	0.375	

**Table 4:** Comparison of clinical symptoms between HSS group (*n* = 43) and control group (*n* = 44)

measurements	***T***_***1***_			***T***_***2***_		
	HSS group	control group	*P*	HSS group	control group	*P*
TMJ Pain (VAS)	1.47 ± 0.89	1.58 ± 0.74	0.476	1.52 ± 0.75	3.84 ± 1.29	0.017*
TMJ Pain (No. of joints)	10/70	13/72	0.882	15/70	45/72	0.004*
TMJ Clicking (No. of joints)	32/70	37/72	0.531	36/70	35/72	0.274

None of HSS, counselling and exercise therapies could alleviate the TMJ clicking. There was a significant TMJ pain relief in both groups. However, pain relief in the HSS group was mainly concentrated at the *T*_*1*_ period, after which there was no statistically significant change at the *T*_*2*_ period. In contrast, the changes of TMJ pain in the control group had statistical significance at both *T*_*1*_ and *T*_*2*_, with pain relief at *T*_*1*_ and pain recurrence at *T*_*2*_. There was no difference between the *T*_*0*_ and *T*_*2*_ periods in TMJ pain in control group. At the *T*_*1*_ period, the VAS value and number of pain joints in HSS and control groups decreased, and the difference between the two groups was not statistically significant. At the *T*_*2*_ period, the VAS value and number of pain joints in the control group increased, which was significantly greater than that of HSS group (*P* = 0.017, 0.004).

### Comparison of TMJ pain between *female and male* in the two groups (Table 5)

**Table 5:** Comparison of TMJ pain between *female* and *male* in the two groups

measurements	HSS group (*n* = male 20 + female 23)					
	*T*_*1*_			*T*_*2*_		
	Ma	Fe	*P*	Ma	Fe	*P*
TMJ Pain (VAS)	1.49 ± 0.93	1.45 ± 0.82	0.476	1.48 ± 0.64	1.73 ± 0.95	0.133
TMJ pain (No. of joints)	4/33	6/37	0.882	6/33	11/37	0.069
measurements	Control group (*n* = male 21 + female 23)					
	*T*_*1*_			*T*_*2*_		
	Ma	Fe	*P*	Ma	Fe	*P*
TMJ Pain (VAS)	1.42 ± 0.68	1.60 ± 1.03	0.654	2.14 ± 1.05	4.33 ± 1.57	0.003*
TMJ pain (No. of joints)	5/35	8/37	0.772	9/35	30/37	0.001*

There were no statistically significant differences in numerical comparisons between all male and female in the HSS group. At the *T*_*2*_ period of the control group, the difference in the number of pain joints and VAS value was statistically significant (*P* = 0.001, 0.003). The VAS value of male (2.14 ± 1.05) was smaller than that of female (4.33 ± 1.57), and the number of pain joints of male (9) was also less than that of female (30).

### MRI descriptions (Table 6)

**Table 6:** Position changes of condyles and discs in post-splint (*T*_*1*_-*T*_*0*_), post-orthodontics (*T*_*2*_-*T*_*1*_) and observation (*T*_*2*_-*T*_*0*_) (male = 20, female = 23,)

Variaties (mm)	*T*_*1*_-*T*_*0*_				*T*_*2*_-*T*_*1*_				*T*_*2*_-*T*_*0*_			
	Male		Female		Male		Female		Male		Female	
	M	SD	M	SD	M	SD	M	SD	M	SD	M	SD
closing position												
Left side												
∠CD-FHVL(°)	-12.17**	6.95	-11.62**	7.64	0.32	0.16	3.58*	1.63	-9.63**	4.33	-7.52**	3.49
C-HRP	-1.84*	0.33	-2.76**	0.45	0.05	0.03	1.34*	0.08	-1.59*	021	-2.87*	0.32
C-VRP	1.42*	0.27	1.69**	0.02	-0.06	0.09	-0.02	0.03	1.30*	0.05	1.60*	0.14
D-HRP	2.87**	1.32	3.98**	0.96	0.28	0.12	-0.33	0.07	2.91*	0.12	3.65*	1.26
D-VRP	-2.31**	0.45	-3.26*	1.37	-0.05	0.08	1.25*	0.36	-1.20*	0.59	-2.94*	1.07
Right side												
∠CD-FHVL(°)	-11.03**	5.94	-13.82**	4.19	0.09	0.47	4.56*	1.59	-9.52**	13.86	-9.20*	14.33
C-HRP	-1.46*	0.95	-2.71**	1.29	0.03	0.51	1.01*	0.33	-1.40*	1.02	-2.35*	0.14
C-VRP	1.47*	0.02	1.76*	0.53	-0.01	0.01	0.02	0.09	1.44*	0.36	1.69*	0.07
D-HRP	2.79*	0.96	3.03**	1.27	-0.09	0.02	-0.05	0.02	2.75*	0.87	2.73*	0.63
D-VRP	-2.09*	0.77	-2.95*	0.33	0.06	0.01	1.02*	0.39	-2.01*	0.48	-2.78*	0.59
opening position												
Left side												
∠CD-FHVL(°)	-1.33	0.36	-0.57	0.41	0.25	0.65	0.38	0.93	-0.24	0.05	-0.95	0.07
C-HRP	-0.12	0.35	-0.06	0.09	-0.02	0.17	-0.02	0.13	-0.02	0.73	-0.07	0.05
C-VRP	0.02	0.67	0.09	0.02	0.02	0.45	0.03	0.52	0.01	0.22	0.09	0.03
D-HRP	0.04	0.05	0.13	0.07	-0.03	0.36	-0.06	0.43	0.08	0.12	-0.07	0.04
D-VRP	-0.66	0.64	-0.29	0.25	0.04	0.25	0.05	0.39	-0.08	0.34	-0.39	0.54
Right side												
∠CD-FHVL(°)	-1.03	1.25	-1.18	0.54	0.31	0.14	1.32	0.52	-0.46	0.25	-0.62	0.13
C-HRP	-0.29	0.17	-0.35	0.08	-0.21	0.07	0.29	0.08	0.89	0.26	0.55	0.18
C-VRP	0.38	0.01	0.09	0.01	0.33	0.20	-0.14	0.07	-0.48	0.15	0.19	0.02
D-HRP	0.72	0.28	0.63	0.08	0.01	0.01	-0.46	0.25	0.11	0.04	0.12	0.08
D-VRP	-0.11	0.13	-0.17	0.05	0.68	0.17	0.35	0.04	-0.55	0.07	-0.01	0.01

*M* is mean, *SD* indicates standard deviation, *Sig* Significance

^*^ *P* < 0.05; ** *P* < 0.01

**Fig. 4:**
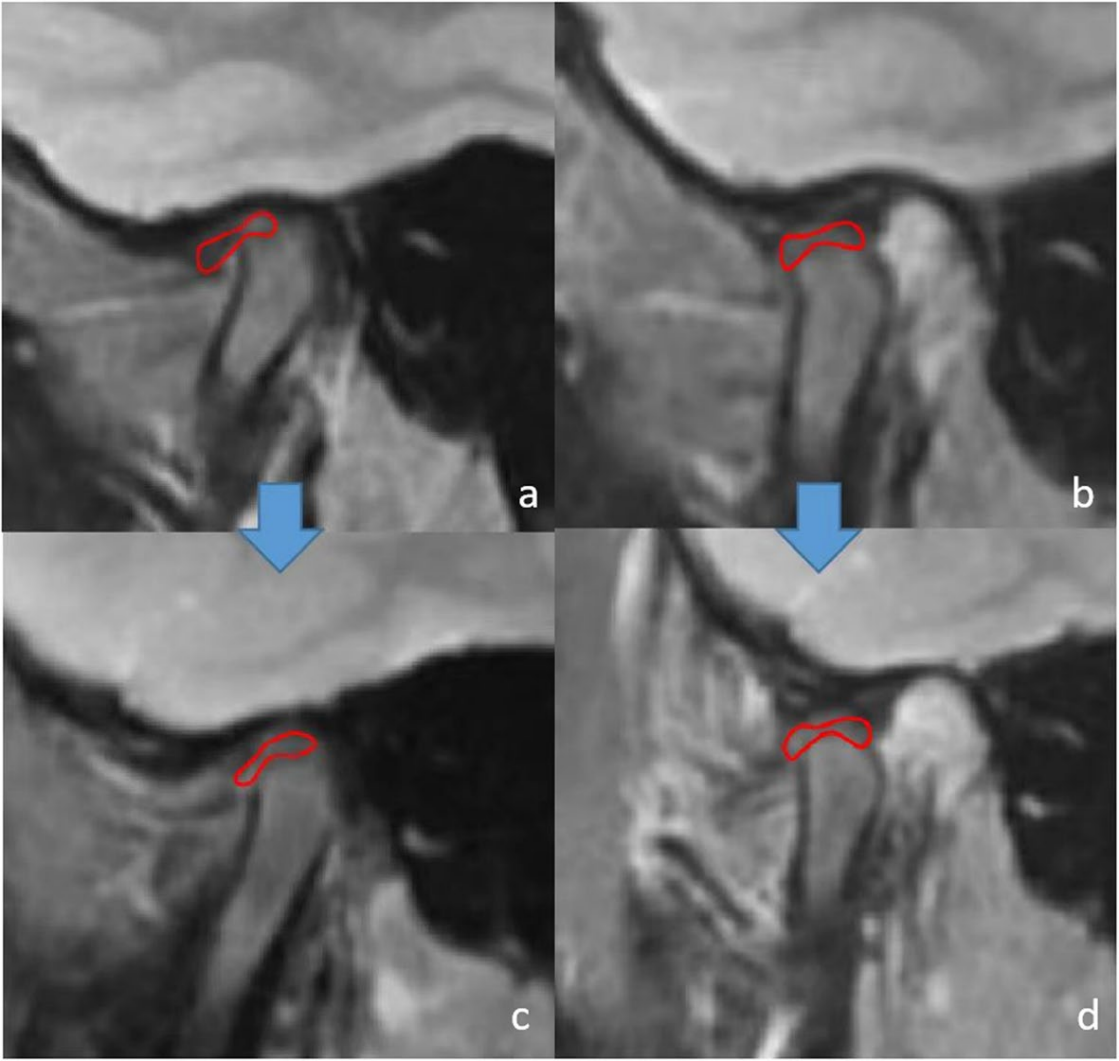
MRIs of typical TMJs before and after HSS therapy. **a** closing position before HSS therapy; **b** opening position before HSS therapy. **c** closing position after HSS therapy; **d** opening position after HSS therapy

Both in male and female, the discs to HRP distance significantly increased and to VRP distance significantly decreased after the HSS treatment in closing position (*P* < 0.05). The disc-condyle angles also decreased (*P* < 0.05). These outcomes indicated that the discs showed posterosuperior movements and condyles showed anterosuperior movements immediately after HSS treatment in all HSS group’s patients. At the *T*_2_ period, female’s disc-condyle angles increased, the discs to HRP distance decreased and condyles to VRP distance increased when compared with the data of *T*_1_ (*P* < 0.05), which meant that there was a tendency to relapse back to discs’ original position. The good news was that the statistically significant difference in (*T*_*2*_*-T*_*0*_) period was still showed in female (*P* > 0.05). In opening position, the movement of discs and condyles had no statistically significant difference, as well as disc-condyle angles. The MRIs in typical cases was showed in the Fig. 4.

## Discussion

### Relationship of orthodontic treatments and TMDs

In normal physiological conditions, there is a harmonious and balanced relationship among condyle, articular disc and fossa. TMDs may occur when this balance is broken [25]. The normal TMJ disc is located between the articular fossa and the condyle. Its posterior articular disc band is located on the transverse crest of the condyle, and the intermediate zone lies interposed between the condyle and the articular eminence [4, 26]. The ideal position of condyles is usually defined by the phrase centric relation (CR): condyles are at the most upper and anterior position of the fossa. At this time, condyles face to the posterior slopes of joint nodules, and articular discs are at a moderate and stable position. CR has nothing to do with occlusion and facial vertical distance, but it is the most stable, comfortable and repeatable physiological position of mandible [27]. While centric occlusion (CO) is a mandible position versus maxilla based on the biting.

Rinchuse, et al. [28] believed that there was no direct relationship between orthodontic treatment and TMDs. They thought TMJ problems could be temporarily ignored before and after orthodontic treatment, especially for patients without joint pain. While others, such as Hudson et al., [29] insisted on the close effect of bites on joints, and they believed that TMDs caused by the improper position of condyle was a problem that needs to be considered and dealt with before orthodontic treatment. At present, it is recognized that orthodontic treatments cannot lead to and cannot treat TMDs, but the reality is that many patients seeking for orthodontic treatment have TMD symptoms such as TMJ pain. For alleviating TMJ pain and discomfort before orthodontic treatment, occlusal splint therapy, counselling, exercises, massage, manual therapy are considered as the main choice treatment because of their low risk of side effects. Besides, some clinicians think occlusal splints, in their experience, can provide a guarantee for the accurate diagnosis and security of TMJs in the people seeking orthodontic treatment.

### Influence of the hard stabilization splint

Both in male and female, clinical examination indicated that joint pain disappeared in two groups at *T*_*1*_ and joint clicking were not eliminated at all in the whole treatment period. However, there was a TMJ pain relapse in control group at *T*_*2*_, especially in female. Which suggested that there was no additional beneficial effect of the use of HSS combined with exercise and counselling therapies in terms of TMJ pain relief in short-term, but after the end of the entire orthodontic treatment, HSS combined with exercise and counselling therapies are better for TMJ pain relief in long-term, especially in female. In addition, in HSS group, MRI showed the disc-condyle angle decreased, and posterosuperior movement of articular discs and anterosuperior movement of condyles occurred at closings after splint treatment. Male had a more stable curative effect than female in terms of the complete orthodontic treatment. All of these suggested that discs, condyles had a tendency to restore the physiologically optimal positions to some extent, and the patients with HSS tended to restore normal disc-condyle relation at closings after splint treatment.

We don't really know exactly what the additional therapeutic mechanism of HSS are when compared with counselling and exercises therapies, but here are some possibilities. (1) The surface of the posterior tooth area of HSS is flat and smooth, which can ensure no interference in mandibular movement and prevent condyles from being locked by ICP (intercuspal position). The anterior tooth area of HSS forms the guiding surface of mandibular anterior and lateral movement, which will ensure a balance in mandibular anterior and lateral movement, and gradually restore TMJ to a comfortable condition [30]. (2) Ettlin [31] suggested that the distance between condyle and articular fossa was sometimes redistributed after splint treatment. This changed space can redistribute the contact area of joint surface, and reduce joint load. So as to cushion abnormal pressure of condylar functional area, release the pressure on disc double plate area, and reduce or even eliminate TMJ injury. (3) The posterior movement of the TMJ disc may be related to the improvement of the disc-condyle relation, so that the TMJ disc can take the advantages of its own elasticity and surrounding attachments, especially the posterior condyle attachment to return to the normal position [32]. Compared with the articular disc itself and other attachments, the posterior condylar attachment is quite different in structure, which still has the property of restoration after being stretched by external force. In the premise of the ADD degree was not serious and the structure of posterior condyle attachments was not damaged, as long as a favorable environment is created, such as using splints to increase joint space and reduce joint pressure, restoration is still possible under the action of elastic fibers of posterior condyle attachments. (3) Other factors can account for the result, such as the increased occlusal vertical distance, eliminated muscle tension, reduced stress of masticatory muscle and TMJ, and inhibited contraction of ascending muscle group.

On the other hand, male had a more stable efficacy in both clinical examinations and MRI results when compared with female’s. We don't really know exactly why things turned out as they did, but we hypothesized some possible reasons for the phenomalea. (1) In clinical practice, normal disc-condyle relations are often not restored, and the temporary stability of disc-condyle relations partly result from the reconstruction of joint discs, which can be helped by HSS. TMJ pain will occur again if such reconstruction—more difficult for female—is not successful. Isberg [33] suggested that the difference results from changes in collagen metabolism associated with the genetic joint laxity. Campos [34] also suggested that the female sexual hormone—oestradiol leads to pro-inflammatory cytokines, as well as aggravating TMJ inflammation. (2) Larger joint spaces were observed in male samples when compared with female samples, especially the superior and posterior spaces in the sagittal view due to the greater thickness of posterior bands of TMJ disks [35, 36]. Kinniburgh [37] also reported that the volume of superior and posterior spaces was associated with disk reduction and pain. So, we hypothesis that smaller articular spaces of female may be an etiologic factor for the higher relapse of TMJ pain on the basis of these findings. (3) The stronger maxillofacial muscle strength of males promoted a more stable soft tissue in the TMJ area. While the weaker muscle strength of young female tended to form a more flexible TMJ disc and ligament, which led to easier displacement of TMJ disc and articular cartilage damage. For the above reasons, the female’s long-term stability of painful TMDs treatment is more difficult than that of male.

There are certain limitations of this retrospective analysis. First, the research has a relatively small sample size due to the strictly controlled inclusion criteria and the required integrity of the research data. Second, it is difficult to use a blind method in retrospective analysis, and the bias is inescapable. Third, the research lack of long-term tracking of clinical efficacy on TMD, which need to be implemented in the future. However, taking the clinical symptoms and MRI results into consideration, we analyzed the influence of gender factor and treatment methods on the clinical efficacy. The useful conclusion could still provide some clinical references.

### Clinical applications

In general, this study suggests such HSS can relieve the pain of masticatory muscles for the orthodontic patients with painful TMD, especially for female. Besides, although there was a slight increase in disc deviation after orthodontic treatment in female, there was still statistical significance in all descriptions between *T*_*0*_ and *T*_*2*_. These results indicates that both in male and female, HSS might improve disc-condyle relations in short-term, but long-term observation and a more adequate statistical analysis are needed.

In addition to treating painful TMDs, some clinicians think HSS also have the function of clarifying mandibular positions and assisting orthodontic diagnosis. In view of the possible relationship between the CO-CR incompatibility and the TMD, it is particularly important for clinicians to determine accurate orthodontic diagnosis under favorable TMJ condition for patients with TMJ symptom. Clinicians’ treatment goal is to achieve coordinated static and functional occlusion, and to limit CO-CR deviation within a certain range as much as possible [38]. Unfortunately, such guiding function we have said about HSS is not scientifically verified, but instead it just represents ideas floating around in the TMD, orthodontic, and restorative communities.

## Conclusion

1. For the orthodontic patients with painful TMD, HSS combined with counselling and exercise therapies before orthodontic treatment could provide pain relief. In addition, male's prognosis is better than female's when without HSS therapy.
2. HSS is helpful to improve the position and relation of discs and condyles. However, there was a tendency to relapse back to discs’ original position, especially in female. Careful and long-term monitoring of the condyle and disc may be needed after splints and orthodontic therapies.

## Data Availability

The data that support the findings of this study are available from Xiangya Stomatological Hospital but restrictions apply to the availability of these data, which were used under license for the current study, and so are not publicly available. Data are however available from the authors upon reasonable request and with permission of Xiangya Stomatological Hospital.

## Abbreviations

MRI: Magnetic resonance imaging
ADD: Anterior disk displacement
ADDR: Anterior disc displacement with reduction
ADDWR: Anterior disc displacement without reduction
TMD: Temporomandibular disorder
TMJ: Temporomandibular joint
CR: Centric relation
CO: Centric occlusion
HRP: Horizontal reference plane
VRP: Vertical reference plane
